# Loss to follow-up in anti-HCV-positive patients in a Brazilian regional outpatient clinic

**DOI:** 10.1590/1414-431X20165455

**Published:** 2016-08-25

**Authors:** L.C. Mendes, S.M. Ralla, A.G. Vigani

**Affiliations:** 1 Universidade Estadual de Campinas, Campinas, SP, Brasil; 2 Ambulatório Municipal de Hepatites Virais, Campinas, SP, Brasil

**Keywords:** Hepatitis C, Follow-up management, Adherence

## Abstract

Loss to follow-up (LF), which refers to patients who started care but voluntary stopped it, is a problem for patients with chronic disease. We aimed to estimate the rate of LF among patients seropositive for hepatitis C virus (HCV) and identify possible demographic and lifestyle risk factors associated with LF. From January 2009 through December 2012, 1010 anti-HCV-positive patients were included in the study. Among participants, 223 (22.1%) met the case definition for LF (more than 1-year elapsed since the last clinical appointment). Among 787 patients who remained in follow-up, 372 (47.2%) were discharged after undetectable HCV RNA, 88 (11.1%) were transferred (and remained on regular follow-up at the destination), and 25 (3.1%) died. According to univariate analysis, male gender, absence of a life partner, black race, psychiatric illness, previous alcohol abuse, previous or current recreational drug use, and previous or current smoking were significantly associated with LF. In multivariate analysis, absence of a life partner (adjusted odds ratio (AOR)=1.44; 95% confidence interval (95%CI)=1.03–2.02), black race (AOR=1.81, 95%CI=1.12–2.89), psychiatric illness (AOR=1.77, 95%CI=1.14–2.73), and the presence of at least one lifestyle risk factor (pertaining to substance abuse) (AOR=1.95, 95%CI=1.29–2.94) were independently associated with LF. Our study provides an estimate of the incidence of LF among anti-HCV-positive patients and identifies risk factors associated with this outcome. In addition, these results can help clinicians recognize patients at risk for LF, who require additional support for the continuity of care.

## Introduction

Hepatitis C is the leading cause of liver disease worldwide and currently 20–30% of individuals with chronic hepatitis C develop cirrhosis. By 2030 this rate is expected to rise to 45% (1,2). Among patients with hepatitis C and cirrhosis, the annual risk of hepatocellular carcinoma (HCC) is 3–5%. With the new direct-acting agents against hepatitis C virus (HCV) infection, sustained virological response (SVR) is possible in over 80% of cases. SVR is associated with increased survival and a significant reduction in complications such as hepatocellular carcinoma and hepatic decompensation (3,4). However, the successful treatment of patients with chronic hepatitis C and prevention of further complications require regular medical follow-up.

Loss to follow-up (LF), which refers to patients who started care but voluntarily stopped it, is a problem for patients with chronic disease. (5,6). There has not been any study in the HCV-infected population in Brazil specifically designed to assess LF rates and few are available worldwide, so the magnitude of this problem remains unknown. In addition, little is known about risk factors associated with LF in patients with HCV infection in outpatient settings.

A clear understanding of how patient characteristics influence LF can assist treatment programs to institute measures for improving patient adherence directed at those with increased risk for interruption of follow-up, such as intensified outreach or follow-up services.

We aimed to identify the rate of LF in outpatients positive for antibodies against HCV (anti-HCV), and demographic and lifestyle risk factors associated with LF.

## Material and Methods

### Study population

Anti-HCV-positive patients from a public regional outpatient clinic for viral hepatitis in the city of Campinas, São Paulo State, Southeast region of Brazil were included in this study. Patients enrolled in the study started care from January 2009 to December 2012. Samples were tested against HCV antibodies using Abbott AxSYM Anti-HCV 3.0 (Abbott Laboratories, Germany). Quantitative serum HCV RNA was assessed by Amplicor HCV 2 (Roche Diagnostics Systems Inc., USA) upon entry into care in order to confirm chronic hepatitis C infection.

### Data collection

We collected patient data using standardized questionnaires indicated by healthcare providers filled during the first visit to the clinic. The questionnaire included demographics information and medical history with special emphasis on comorbidities and lifestyle. Lifestyle encompassed previous or current alcohol abuse, previous or current recreational drug use, and tobacco smoking. Alcohol abuse was defined as seven or more drinks per week. Current alcohol abuse, illicit drug use, and smoking were considered active, if it occurred during the six months prior to study enrollment, and previous, if discontinued before that period. In case of LF, a second questionnaire was used for collecting information regarding reasons for care interruption. All information collect was entered into EPI INFO 2000 (Centers for Disease Control and Prevention, USA).

### Definition of loss to follow-up

We defined LF as patients with more than 1 year since last clinic appointment. When patients presented more than one interruption during the study period, only the first episode was considered for analysis. LF rate was calculated using the number of LF events divided by the total number of patients who started treatment during the study period. Patients transferred to other health facilities were not considered as LF.

When patients interrupted clinic visits for over 12 months, a recall was attempted through available contact information. We also reviewed medical records to locate information related to death, hospital transfers, and any other relevant data.

### Factors associated with LF

We compared the characteristics of patients who interrupted care with patients who attended the clinic regularly during the study period to identify factors associated with follow-up interruption. Demographic data included age at enrollment, gender, and race. Lifestyle data included previous or current alcohol abuse, recreational drug use, and tobacco smoking.

### Statistical analyses

Continuous variables are reported as means and standard deviations, and categorical variables as frequencies, unless otherwise stated. The *X* ^2^ test was used for nominal categorical variables. Analysis of variance (standard or nonparametric, as appropriate) was used to test associations between continuous and categorical variables. Variables for which an association was suspected in the univariate analysis (i.e., P≤0.20) were included in a stepwise logistic regression model. All analyses were performed with EPI INFO 2000. A significance level of 5% (P<0.05) was used throughout.

### Ethical considerations

This study is the result of a retrospective evaluation of medical records of patients treated at a municipal clinic under routine care, and it was approved by the Ethical Committee of the "Centro Infantil de Investigações Hematológicas Dr. Domingo A. Boldrini" (process #39002414.5.0000.5376). Patient informed consent was not deemed necessary for this study.

## Results

Between March 1 and December 31, 2012, 1010 anti-HCV-positive patients started care in this outpatient clinic. Patient characteristics are show in Table 1. Among all patients, 55.1% were male and the mean age at the beginning of follow-up was 49 years. Information about race was available for 987 (97.7%) patients of which 11.1% were black. Almost 45% (444/992) of patients reported not having a life partner, such as spouse or domestic partner. In regards to comorbidities, information was available for 943 patients, of which 136 (14.4%) reported diabetes and 40 (13.4%), psychiatric disorders. Moreover, self-reported information on lifestyle risk factors was available for 979 patients with 657 (67.1%) who had at least one lifestyle risk factor. The most common lifestyle risk factor was current or previous alcohol consumption (n=495; 50.6%) and current tobacco smoking (n=442; 41.4%). Among all enrolled patients, when LF occurred, 576 (57.0%) had detectable serum HCV RNA, 372 (36.8%) had undetectable serum HCV RNA, and the remaining 62 (6.1%) patients were lost to follow-up before RNA testing. Specific treatment for HCV had not yet been prescribed for any patient when LF occurred.

**Table:**
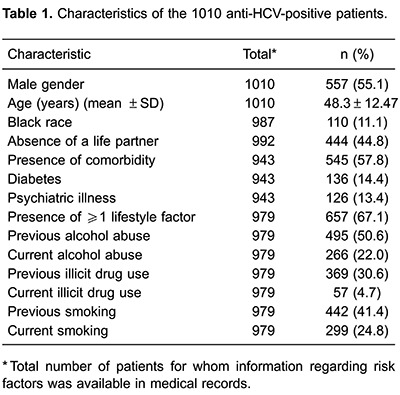

**Figure 1:**
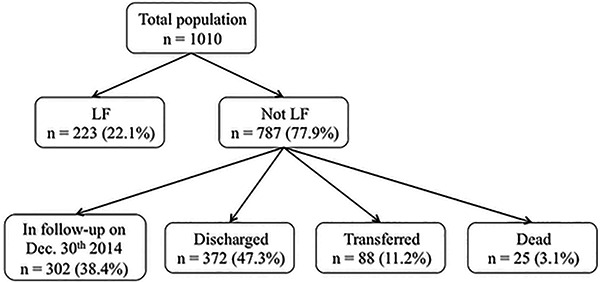
Outcomes of the study population. LF:lost to follow-up.

The outcomes of the study population are showed in Figure 1. Case definition for LF was met in 22.1% of patients (223 of 1010) and of these, 5 died after LF. Among the 1010 patients, 787 (77.9%) did not meet the case definition of LF. Among these, 47.2% were discharged after undetectable levels of HCV RNA, 88 of 787 (11.2%) were transferred to other facilities, and 25 (3.1%) died. The median duration of follow-up for lost patients was 4 months and 37.7% of those patients interrupted care with less than 30 days after starting it.

According to univariate analysis, gender, absence of a life partner, black race, psychiatric illness, and at least one lifestyle risk factor were significantly associated with LF (Table 2). Single black male patients were more likely to experience LF. Among lifestyle factors, previous alcohol abuse, previous and current illicit drug use, and previous or current tobacco smoking were associated with LF. In the multivariate analysis, absence of a life partner, black race, psychiatric illness and substance abuse were independently associated with LF (Table 3).

**Table:**
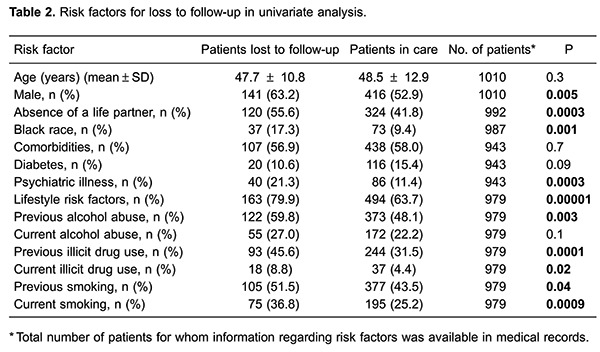

**Table:**
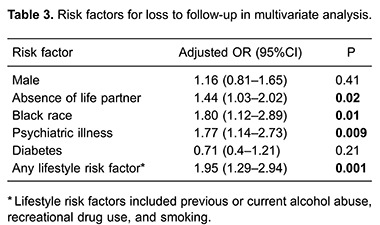

We were able to locate 104 (46.6%) of the 223 patients lost to follow-up and only 33 (14.8%) returned to follow-up visits. The most frequent reason for LF stated by patients was incompatibility between work hours and scheduled clinical appointments, followed by incarceration (Table 4). However, for 122 (54.7%) patients no reason for losing follow-up could be provided.

**Table:**
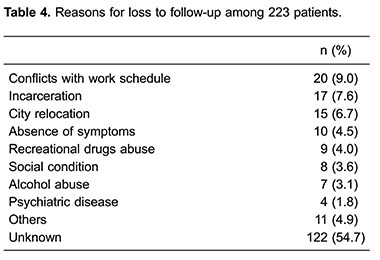

## Discussion

With recent advances in anti HCV treatment, SVR can be reached in 80–90% of patients (7). In this new scenario, it is now possible to envision a drastic reduction of HCV prevalence, but major challenges remain. One of them, according to our findings, is LF. Among anti-HCV-positive patients, we found a LF rate of 22.1%, and male, absence of a partner, black race and the presence of one lifestyle risk factor were independently associated with LF. In addition, almost 40% of patients were lost to follow-up within less than one month after starting care. Finally, less than 15% of patients lost to follow-up returned to the clinic after being actively recalled.

There have been few studies evaluating LF and non-adherence to care in the HCV infected population. Notably, most data regarding non-adherence and patient compliance are related to treatment-associated factors, and are not regarded as a primary outcome (8–11). For this reason, many patients who quit follow-up before complete staging and treatment indication are not included in those studies. Therefore, evaluating patient adherence to care in the initial steps of HCV evaluation is paramount for a more complete understanding of the problem.

Before therapy has been indicated, treatment eligibility is directly influenced by patient compliance with follow-up. In Brazil, eligibility for treatment has been evaluated in HIV/HCV co-infected patients and non-adherence to therapy was found to be responsible for 31.4% of ineligibility for HCV treatment (12). According to our findings, patients are lost to follow-up soon after initiating care, which has also been observed in previous studies (13,14). A Greek study found that 27% of anti-HCV-positive were lost to follow-up before HCV RNA testing, and the majority of patients who did not receive treatment were lost to follow-up (15). When evaluating LF as a risk factor for treatment discontinuation and, similarly, for treatment failure, observational studies have found that non-adherence was directly associated with non-response to therapy in 18 to 29% of patients (8,9,16–18). Our results corroborate those findings and call attention to LF as an important reason for treatment ineligibility.

Non-adherence to care is a known issue for chronic illnesses. In terms of chronic, mostly asymptomatic infections, HIV and HCV share some similarities, especially considering the need for close monitoring and follow-up. In our cohort, the LF was similar to that observed around the world among HIV-positive patients. Among HIV-positive patients, the LF rates range from 13.4 to 25% (13,19–21). In Brazil, among HIV-positive patients, the LF rates ranged from 17.6 to 23.9% (22,23). The great variability observed in HIV-positive populations can be related to the lack of standardized definitions on follow-up time, which ranges from 3 months in some studies to up to 12 months in others (22,24–27). Secondly, the different population characteristics in the various regions of the world that could impact patients' ability to consistently maintain access to health care (28), can influence LF rates. It is necessary to consider that these differences may also be true for HCV infection.

Establishing risk factors associated with LF has the potential to identify patients that require additional efforts to remain in follow-up. Similar to our findings, previous studies have also found a correlation between both alcohol consumption and psychiatric illness with ineligibility for treatment and also with treatment failures (10,12,29). The association found between absence of a partner and LF is difficult to be fully understood. A possible explanation is that patients without partners do not possess sufficient emotional and social support systems. Finally, a chronic physical disease not only has direct consequences for the patient, but can also disrupt the normal life of the healthy partner (30).

According to the NHANES report, the HCV prevalence is 14% among black people and 1.6% in the general population in the USA, and black people with chronic hepatitis C have higher age-adjusted mortality rates from cirrhosis and CHC than non-Hispanic whites with chronic hepatitis C (31). According to our findings, black race was a recognizable risk factor for LF. It is possible that the high rate of black patients who are lost to follow-up prior to fibrosis staging and treatment indication may be responsible for some of the high CHC incidence in this group, and they might not be represented in studies of treatment response.

In patients with hepatitis C, the association between psychiatric disorders and LF is common. Patients infected with HCV have a high prevalence (50%) of psychiatric disease. In addition, the lifetime prevalence of psychosis, anxiety, substance abuse, and personality disorders are all higher among patients with HCV than the general USA population (32). The association between lifestyle factors (excess alcohol consumption, tobacco smoking or recreational drug use) could also be associated with impulsive personality and hopelessness (33). In that sense, depression is the most commonly diagnosed mood disorder in HCV-infected patients and has been extensively associated with barriers to compliance with care and treatment adherence (12,34–36).

We were able to locate only 46.6% of the patients lost to follow-up. This finding suggests that efforts should be made to encourage patients to provide as many personal contacts as possible, and additional information on family members or friends. In addition, for 54.7% of LF patients no reason for non-compliance with care could be found. This demonstrates that LF is a complex multifactorial process and understanding patient-associated reasons for non-compliance can present a challenge. Based on our findings, we suggest that recalling patients is necessary but not sufficient to ensure continuity of care. Similar studies in the HIV-infected population showed that 4 to 28% of patients returned to care after recall (37,38).

Our study has some limitations. We were not able to examine social characteristics, such as homelessness and unemployment states because these variables were not systematically reported in the medical records. Finally, data on telephone number and name of primary care physician were retrospectively collected after reviewing patient's charts, and might lack precision.

In conclusion, our study provides an estimated incidence of LF among anti-HCV-positive patients and identifies risk factors associated with this outcome. Importantly, our results comprise LF data from patients at the first stage in the HCV chain of care. In addition, our study can help clinicians recognize patients at risk for non-compliance and who require additional support for retention of care. Longitudinal studies are urgently needed to better identify these patients as well as variables that affect their retention in care. A clear understanding of how patient characteristics influence treatment adherence can assist the development of better strategies to improve patient outcomes.
